# Real-time device-scale imaging of conducting filament dynamics in resistive switching materials

**DOI:** 10.1038/srep27451

**Published:** 2016-06-07

**Authors:** Keundong Lee, Youngbin Tchoe, Hosang Yoon, Hyeonjun Baek, Kunook Chung, Sangik Lee, Chansoo Yoon, Bae Ho Park, Gyu-Chul Yi

**Affiliations:** 1 Department of Physics and Astronomy, Institute of Applied Physics and Research Institute of Advanced Materials (RIAM), Seoul National University, Seoul 151-747, Korea; 2 Department of Physics, Konkuk University, Seoul, 143-701, Korea

## Abstract

ReRAM is a compelling candidate for next-generation non-volatile memory owing to its various advantages. However, fluctuation of operation parameters are critical weakness occurring failures in ‘reading’ and ‘writing’ operations. To enhance the stability, it is important to understand the mechanism of the devices. Although numerous studies have been conducted using AFM or TEM, the understanding of the device operation is still limited due to the destructive nature and/or limited imaging range of the previous methods. Here, we propose a new hybrid device composed of ReRAM and LED enabling us to monitor the conducting filament (CF) configuration on the device scale during resistive switching. We directly observe the change in CF configuration across the whole device area through light emission from our hybrid device. In contrast to former studies, we found that minor CFs were formed earlier than major CF contributing to the resistive switching. Moreover, we investigated the substitution of a stressed major CF with a fresh minor CF when large fluctuation of operation voltage appeared after more than 50 times of resistive switching in atmospheric condition. Our results present an advancement in the understanding of ReRAM operation mechanism, and a step toward stabilizing the fluctuations in ReRAM switching parameters.

Resistive random access memory (ReRAM) is considered one of the promising non-volatile memory devices to overcome the limitations of Si-based flash memory due to its high operating speed, low power consumption, and high scalability^1^,^2^,^3^. However, fluctuations in its switching parameters during operation remain a critical weakness that leads to device failures^4^. To enhance the stability of the operation parameters, it is important to understand the switching mechanism of ReRAM devices. Recent studies using transmission electron microscopy (TEM)^5^,^6^,^7^,^8^,^9^ and conductive atomic force microscopy (C-AFM)^10^,^11^,^12^ have suggested that the formation of conducting filaments (CFs) in bulk or oxygen migration at the interface results in resistive switching. In particular, *in situ* TEM studies have shown the time evolution of CFs, which facilitates a microscopic understanding of the resistive switching mechanism^8^,^9^. Nonetheless, the problem of the instability of switching parameters remains unsolved because the instability is thought to involve the dynamics of single or multiple CFs at the device scale, whereas TEM or AFM studies are unsuited for simultaneously making real-time measurements at multiple spots over a large area. Here, we introduce a real-time device-scale imaging of the configuration of CFs by combining ReRAM and a light-emitting diode (LED) in a hybrid device, which offers a comprehensive understanding of the origin of resistive switching fluctuations by monitoring the dynamics of multiple CFs in action during the ReRAM device operation.

## Materials and Methods

### Materials

**Figure 1:**
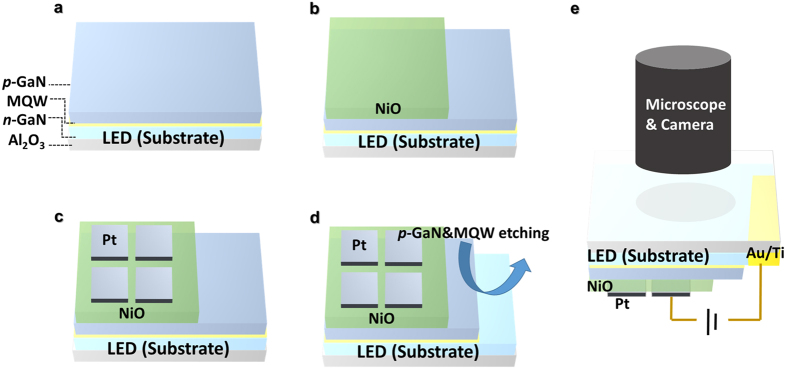
Schematics of device fabrication process and measurement set-up. (**a–d**) Pt and NiO thin film were deposited on the LED substrate. (**e**) The ReRAM/LED hybrid structure was flipped to measure light emission behavior through the microscope and camera.

A GaN thin film was deposited on an Al_2_O_3_ (0001) substrate at three temperature steps: 540 °C for 2 min, 970 °C for 15 min, and 1080 °C for 1 h, at flow rates of 15–30 sccm of trimethylgallium (TMGa). The substrate was then heated at 1050 °C for 1 min with H_2_ and NH_3_, and a GaN-based *p-n* junction LED structure with In_1−*x*_Ga_*x*_N/GaN multi quantum wells (MQW) was grown heteroepitaxially on the surface of the GaN thin film. First, a Si-doped *n*-GaN layer was deposited at flow rates of 15–30 sccm for TMGa and 1–3 sccm for ditertiarybutyl silane. Then, eight-period In_1−*x*_Ga_*x*_N/GaN MQWs were grown at 760 °C and 850 °C, respectively. Finally, a Mg-doped *p*-GaN layer was deposited on the top of the GaN quantum barrier layer at 1000 °C, using biscyclopentadienyl magnesium as the doping source. Schematic structures of a ReRAM/LED hybrid device are shown in Fig. 1. To fabricate a ReRAM/LED hybrid device, a 35-nm-thick polycrystalline NiO film was prepared by DC reactive sputtering on a *p*-GaN/MQW/*n*-GaN/GaN/Al_2_O_3_ LED substrate at 500 °C and 1.5 mTorr pressure in an Ar + O_2_ gas mixture (7% O_2_). Pt top electrodes with dimensions of 100 × 100 μm^2^ were deposited and patterned using DC reactive sputtering and a conventional lift-off process, respectively, under the same conditions as described in our previous studies^13^,^14^,^15^. Lithography and BCl_3_ reactive ion etching were used for the exposure of *n*-GaN layers. Au/Ti (20/20 nm) bilayers were deposited on the *n*-GaN surface and annealed to make ohmic contacts and reduce contact resistance of the electrodes.

### Electrical measurements

*I–V* characteristic curves were measured using a Keithley 2400 sourcemeter in direct voltage scanning at room temperature and atmospheric pressure. Real-time video of light emitting from the ReRAM/LED hybrid device was acquired using an optical microscope and commercial digital camera; the time resolution of the video was 0.04 s. All of the video files are introduced in supplementary video 1 and 2.

## Results and Discussion

Figure 1 presents a schematic of device fabrication and measurement set-up of the ReRAM/LED hybrid device. As the resistive memory material, a NiO thin film was deposited on a GaN-based LED. Then, Pt top electrodes were fabricated on the NiO/LED substrate. To access the underlying *n*-GaN as the bottom electrode, *n*-GaN layers were exposed by lithography and reactive ion etching, and Au/Ti were deposited by thermal evaporation (see Materials in Materials and Methods section). To observe the light emission behavior from the back side of the LED, the ReRAM/LED hybrid structure was flipped toward the microscope and camera.

**Figure 2:**
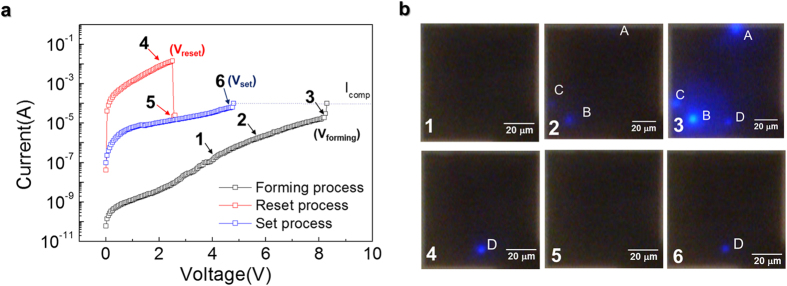
*I–V* characteristic curves of the ReRAM/LED hybrid structure during forming and reset/set processes and corresponding light emission images. (**a**) *I–V* characteristic curve and (**b**) light emission images of a ReRAM/LED hybrid device during the forming and conventional reset/set processes. Light-emission images were obtained when the *I–V* characteristic curves reached the points marked with arrows and numbers during the resistive switching. A compliance current of *I*_comp_ = 10^−4^ A was set to prevent breakdown during the forming and set processes.

The forming process of the hybrid device was first investigated using this set-up, during which a conducting path was created between the top and bottom surfaces of the NiO thin film upon application of a sufficiently large electric field. In Fig. 2a, the current *–* voltage *(I–V)* characteristic curve of the forming process shows clearly that sweeping the voltage to positive values above a forming voltage (*V*_forming_ = 8.20 V), with an appropriate compliance current to prevent breakdown, resulted in an abrupt increase in the current level. This voltage sweep above *V*_forming_ modified the state of the NiO film to a non-volatile low-resistance state (LRS). During the forming process, the light emission of the ReRAM/LED hybrid device was investigated in real time, which revealed the anomalous emergence of multiple current conduction spots. As shown in Fig. 2b–1,2, three light spots appeared, at spots A, B, and C, as the voltage was swept near point 2 (6.50 V, 4.12 μA). However, at point 3 (8.20 V, marked as *V*_forming_, 18.9 μA), where the current level increased abruptly, an additional light at spot D was observed. This suggests that the light at spot D, rather than that at spots A, B, and C, is related to the forming process. Meanwhile, the diameters of the light spots are around 5 μm, much larger than the current path through the CF with its typical diameter of around 10 nm^7^,^8^, resulting presumably from divergence of the emitted light.

**Figure 3:**
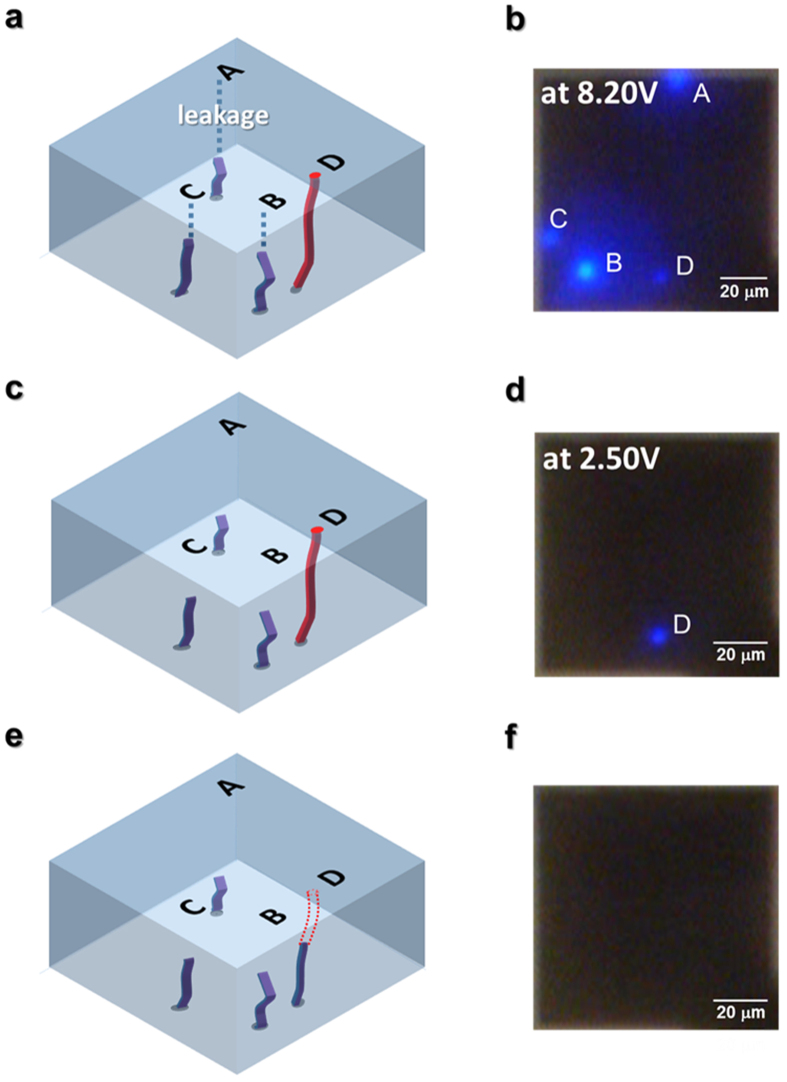
Illustration of CF configuration in NiO thin film. (**a**) forming, (**c**) LRS, and (**e**) HRS states, and (**b,d,f**) their corresponding light emission images.

**Figure 4:**
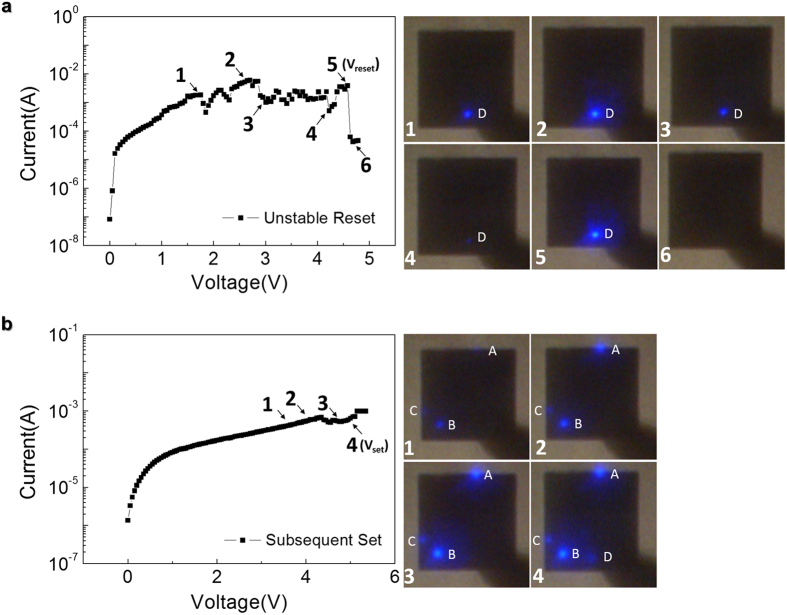
*I–V* characteristic curves of the ReRAM/LED hybrid structure during unstable reset and its subsequent set and corresponding light emission images. (**a**) an unstable reset process and (**b**) its subsequent set process.

The role of spot D was further confirmed by investigating the *I–V* characteristic curve and light emission in the LRS of the ReRAM/LED hybrid device. Sweeping the voltage from 0 V again after the forming process, as shown in Fig. 2a (red curve), the current conduction was many orders of magnitude larger than the initial sweep (black curve), signifying a LRS. During this step, only one light spot was observed at D, as shown in Fig. 2b–4, indicating that, of the four lighting spots A, B, C, and D in Fig. 2b–3, the resistance between the top and bottom surfaces of the NiO thin film was lowest at spot D. This indicated that only spot D developed a fully connected metallic conduction channel between the two surfaces, which is responsible for the forming behavior, while spots A, B, and C were likely due to leakage currents, as will be seen in more detail in subsequent experiments. Under this scenario, it may appear odd that the light intensity is weaker at spot D than at the other spots in Fig. 2b, despite hosting a much larger current than the other spots (note the logarithmic scale in Fig. 2a). This is due to the high temperature, near 800 °C, of the CF at spot D caused by Joule heating during CF formation^16^, which locally degraded the light emission efficiency of the LED.

**Figure 5:**
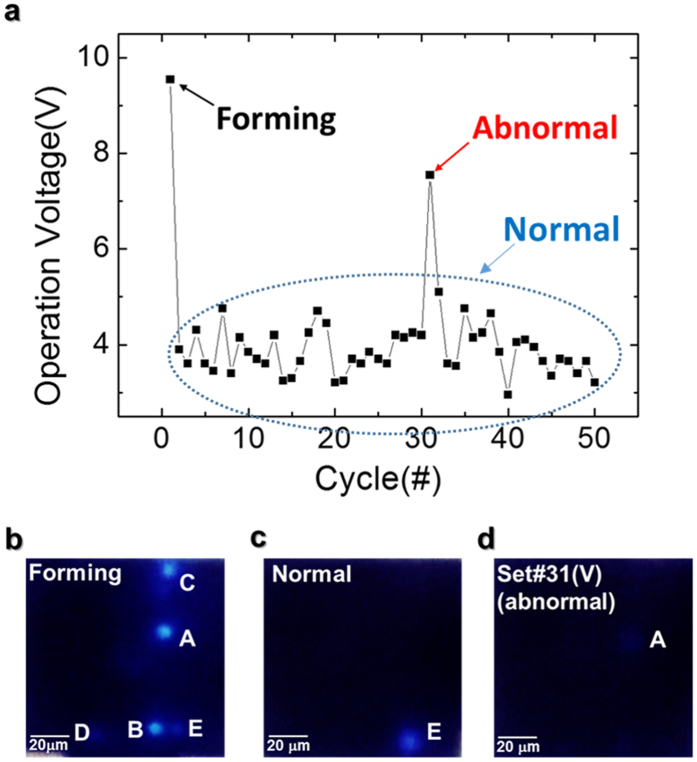
Fifty resistive switchings and real-time light emission. (**a**) *V*_set_ versus switching cycle and (**b*****–*****d**) light emission images obtained at several set processes during 50 resistive switchings. A new ReRAM/LED hybrid device was used for this experiment.

Further sweeping of the voltage resulted in unipolar resistive switching behaviors between LRS and a high resistance state (HRS), where the switching from LRS to HRS is called a reset process, and from that HRS to LRS, a set process. During the reset process (Fig. 2a, red curve), according to the light emission from spot D even at low applied voltage marked as 1, we assume that the conducting filament (CF) at spot D is connected during the reset process, which results in flowing most of current via CF at spot D in contrast to the forming process. The current in LRS increases with the applied voltage up to *V*_reset_, after which a step-like drop in current is seen, along with a simultaneous dimming of the light at spot D (Fig. 2a–5), indicating a switch from LRS to HRS. Conversely, sweeping the voltage from 0 V again after a reset process results in a set process, where the current in HRS abruptly increases above *V*_set_. Simultaneously, the light at spot D turns on (Fig. 2a–6), indicating a switch from HRS to LRS. These processes can be repeated multiple times to create 0 and 1 states of a non-volatile memory.

To examine the dynamics of light emission spots during resistive switching, we suggest the model illustrated in Fig. 3, which is based on observations of randomly grown CFs demonstrated by TEM and AFM in previous reports, namely the surface of the oxide thin film studied using AFM^10^,^11^,^12^ during resistive switching, and disconnected CFs near the top electrode observed at LRS using TEM^7^. Figure 3a,b, respectively, show the configuration of CFs and their light emission image during the forming process, depicting three minor CFs (spots A, B, and C) and a major CF (spot D). Light emissions at minor CFs observed at voltages as small as 6.50 V before the forming process at 8.20 V can be ascribed to leakage currents originating from insufficiently formed CFs and/or structural defects, which will be discussed in more detail in relation to the fluctuation behaviors to be seen shortly. These conduction spots do not accompany sudden jumps in current or light emission intensity, indicating that the overall conduction path remains non-metallic. After the major CF connects the top and bottom surfaces of the NiO thin film under an applied electric field due to the forming process, the ReRAM device switches to LRS. Then, carriers are injected into the LED via the major CF, and light is emitted even at a low applied voltage near 2.50 V, as illustrated in Fig. 3c,d. After a reset process, the major CF is ruptured, and the ReRAM device switches to HRS, as illustrated in Fig. 3e. Due to the ruptured CF, carrier injection into the LED is reduced markedly, and light emission disappears, as shown in Fig. 3f.

Repeating the switching experiments further, we were able to observe two different types of occasional ReRAM device fluctuation behaviors and to get glimpse the nature of the anomalous current conduction at the minor CFs. The first type of fluctuation involves an unstable reset process, as shown in Fig. 4a, where the major CF blinks fiercely during the voltage sweep in LRS and switches to HRS at an unusually high *V*_reset_ of 4.50 V. This is presumably because the major CF goes through soft ruptures and simultaneous formations due to the accumulated stress of repeated switching. Moreover, the subsequent set process, shown in Fig. 4b, reveals that the minor CFs can now emit light at a voltage as low as 3.60 V (as opposed to 6.50 V during the forming process, Fig. 2b–2). Meanwhile, if the light from spot A, B, and C are emitted by current flow through completely formed CFs, turn-on voltages of the spot A, B, and C would not be affected by previous unstable resistive switching process and they should be observed at the same voltage with in case of Fig. 2b–2. In more detail, if the origin of the light emission from spot A, B, and C are regarded as the leakage currents originating from incompletely formed CFs, decrease of the turn on voltages is able to be emerged by decrease of the distance between incompletely formed CFs and top electrode (increase of the leakage currents even at low voltage) resulting from repetition of soft rupture and soft formation processes introducing blinking of the light at spot D. This suggests that conducting paths from resistive switching were involved at spots A, B, and C, albeit in incomplete form (which is why we call them minor CFs), which grew over time during the switching processes to lower the threshold voltage for the light emission of the hybrid ReRAM/LED stack at those spots.

The second type of fluctuation involves a set process in which the major CF becomes stressed and causes switching from a different CF. Using a new device, we performed 50 consecutive resistive switchings and light emission imaging to investigate the unstable resistive switching characteristics. Figure 5a shows the *V*_set_ of the ReRAM/LED hybrid device in these measurements. At a forming voltage of 9.55 V, many light emission spots appeared, as shown in Fig. 5b, similar to the behavior seen in Fig. 2. After the forming process, normal set operations showed consistent set switching at voltages around 4.00 V associated with the major CF at spot E as shown in Fig. 5c. However, as is often observed in repeated ReRAM switching measurements^4^, we found a sudden increase in *V*_set_ at cycle #31 (marked as Abnormal), accompanied by a change in light emission configuration. For this cycle, spot E was dark, but there was a faint emission at spot A, as shown in Fig. 5d. This was attributed to the deterioration of the major CF at spot E and subsequent connection of an alternative minor CF at spot A at a higher voltage. Meanwhile, except in case of cycle #31, light emission is observed at spot E for every cycles. Individual *I–V* characteristic curves measured before and after cycle #31 (see Figure S1) show significantly different current levels, supporting this explanation.

## Conclusion

In conclusion, we propose a new hybrid structure composed of a ReRAM and LED that enables monitoring of the time evolution of CF configurations at the device scale during resistive switching. In contrast to former studies using *in situ* TEM or AFM, the change in CF configuration across the whole device area was observed in real time. Using the ReRAM/LED hybrid structure, conventional form/set/reset processes, as well as the fluctuation behaviors during set and reset processes, were investigated. In particular, we observed that minor CFs were formed earlier than the major CF contributing to the resistive switching behavior. Furthermore, we investigated the unstable reset process accompanied by the growth of minor CFs and the fluctuation among set processes, where the configuration of the major CF was changed when a large fluctuation in operation voltage appeared. We believe that these findings further the understanding of the fluctuation behaviors in ReRAMs and present a step toward enhancing the reliability of ReRAM devices.

## Additional Information

**How to cite this article**: Lee, K. *et al.* Real-time device-scale imaging of conducting filament dynamics in resistive switching materials. *Sci. Rep.* **6**, 27451; doi: 10.1038/srep27451 (2016).

## Supplementary Material

Supplementary Information

Supplementary Video 1a

Supplementary Video 1b

Supplementary Video 1c

Supplementary Video 1d

Supplementary Video 1e

Supplementary Video 2a

Supplementary Video 2b

Supplementary Video 2c

Supplementary Video 2d

Supplementary Video 2e
