# Supplementary material for: Real-time device-scale imaging of conducting filament dynamics in resistive switching materials
Source: Sci Rep. 2016 Jun 7;6:27451. doi: 10.1038/srep27451 (PMC4895219; doi:10.1038/srep27451)
Supplement: Supplementary Information [file srep27451-s1.doc]

Supplementary Information for

Real-time device-scale imaging of conducting filament dynamics in resistive switching materials

Keundong Lee^1^, Youngbin Tchoe^1^, Hosang Yoon^1^, Hyeonjun Baek^1^, Kunook Chung^1^, Sangik Lee^2^, Chansoo Yoon^2^, Bae Ho Park^2^* and Gyu-Chul Yi^1^*

^1^ Department of Physics and Astronomy, Institute of Applied Physics and Research Institute of Advanced Materials (RIAM), Seoul National University, Seoul 151-747, Korea

^2^ Department of Physics, Konkuk University, Seoul, 143-701, Korea

*Correspondence and requests for materials should be addressed to B. H. P. (email: [baehpark@konkuk.ac.kr](mailto:baehpark@konkuk.ac.kr)) and G.-C. Y. ([gcyi@snu.ac.kr](mailto:gcyi@snu.ac.kr))

***I–V* characteristic curves and light emission images of 50 cycles of resistive switching**


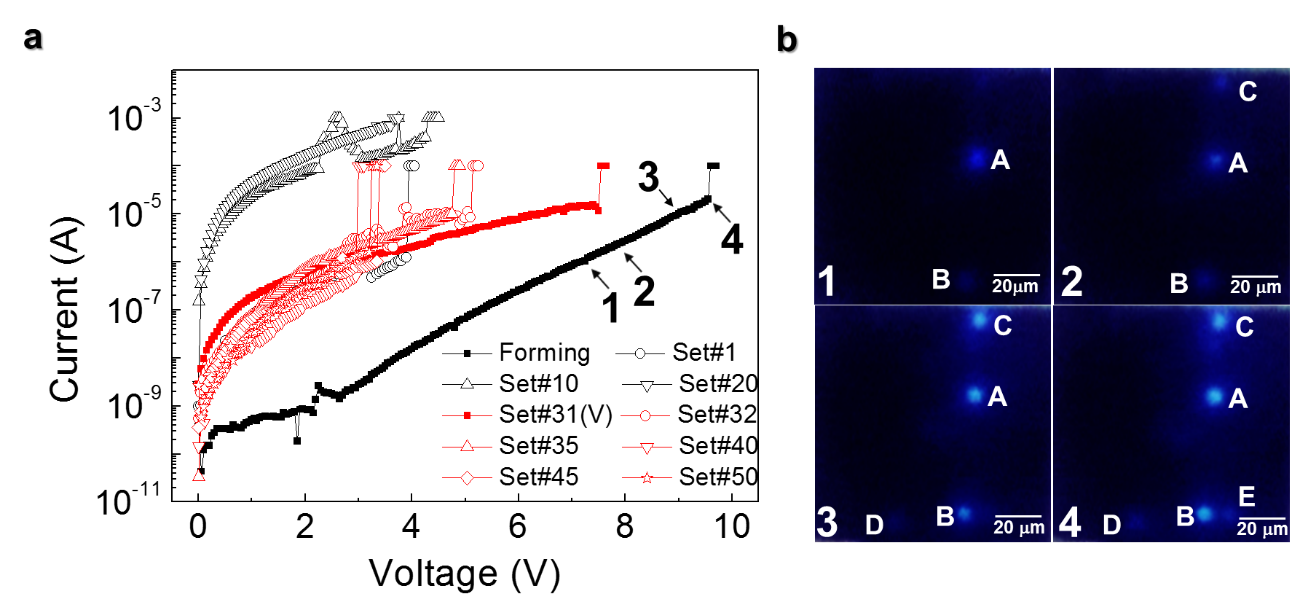
 **Figure S1**. (a) *I–V* characteristic curves of the forming process and 50 cycles of set processes for the ReRAM/LED hybrid device used in Figure 5. (b) Light emission images during the forming process.

Before studying the relationship between the *V*_set_ fluctuation and the conducting path configuration in Figure 5, the forming process and light-emission images were analyzed, as shown in **Figure S1**. Similar to Figure 2a, multiple minor CFs were first observed as the applied voltage was increased. When the current showed an abrupt increase at point 4, light at spot E appeared suddenly; we identified this as the major CF responsible for the resistive switching for this device. For the subsequent set processes in Figure S1a, open black symbols mark set processes before cycle #31, solid red symbols mark set process at cycle #31, and open red symbols mark set processes after cycle #31. Meanwhile, at cycle #10 and #20, current level temporarily increases before the applied voltage reaches at *V*_set_. This incident behavior has been reported as unstable resistive switching regarding as weakness of the ReRAM.^1, 2^ As discussed in Figure 5, at cycle #31, the major CF at spot E was stressed and caused an anomalous switching to occur, with a faint light emission at spot A instead, accompanied by an abnormally large *V*_set_. After this behavior, the switching CF returned to spot E, and *V*_set_ was stabilized to levels similar to those used before. However, we observed that the overall current level of the set operations changed significantly, possibly indicating a degradation of the major CF.

***I–V* characteristic curves of the individual LED and ReRAM which have used in our experiment.**

**Figure S2.** *I*–*V* characteristic curves of the individual LED and ReRAM which have used in our experiment.

Au(20nm)/Ni(20nm) contacts were used as the top electrodes of the LED and Pt/NiO was deposited to fabricate conventional LED and ReRAM structure, respectively. For the conventional LED, its I–V characteristic curve shows a typical rectifying behavior at 3V. Meanwhile, our hybrid device of ReRAM and LED shows a unipolar switching behavior at lower voltages (*V*_forming_ : 1.75V, *V*_reset_ : 0.45, and *V*_set_ : 1.55V).

**Supplementary Video Legends**

**Supplementary Video 1.** Real time videos of the light emission during the (a) forming, (b) reset, (c) set, (d) unstable reset, (e) its subsequent set processes.

**Supplementary Video 2.** Real time videos of the light emission during the fifty resistive switchings especially for (a) forming process, (b) normal set process, (c) normal set process at 30^th^ resistive switching, (d) abnormal set process at 31^st^ resistive switching, and (e) normal set process at 32^nd^ resistive switching. For clear observation, we controlled the contrast of the captured images as displayed in Figure 5 and Figure S1.

^1^ Yu, D. *et al.* Self-compliance unipolar resistive switching and mechanism of Cu/SiO_2_/TiN RRAM devices. *2012 IEEE Silicon Nanoelectronics Workshop (SNW)*. (2012).

^2^ Rahaman, Sheikh Ziaur, and Siddheswar Maikap. Comparison of resistive switching characteristics using copper and aluminum electrodes on GeO_x_/W cross-point memories. *Nanoscale Res Lett* **8,** 1 (2013).
